# Cerebral differences between roller and speed skaters: preliminary evidence for roller-to-ice talent transfer

**DOI:** 10.3389/fpsyg.2026.1778839

**Published:** 2026-07-17

**Authors:** Qing Yan, Keying Zhang, Ling Jiang, Yuyan Wang, Yihua Cao, Chunmei Cao, Dong Zhang

**Affiliations:** 1 Institute of Artificial Intelligence in Sports, Capital University of Physical Education and Sports, Beijing, China; 2 Department of Physical Education, Southeast University, Nanjing, China; 3 Division of Sports Science and Physical Education, Tsinghua University, Beijing, China

**Keywords:** brain plasticity, magnetic resonance imaging, neural mechanism, skating, talent transfer

## Abstract

**Objectives:**

Talent transfer (TT) from roller skating to speed skating is a recognized pathway for developing elite athletes. This study aimed to identify the sport-specific cerebral characteristics in these athletes by comparing their brain structure and function, thereby providing preliminary neuroimaging evidence relevant to “roller-to-ice” TT.

**Methods:**

We acquired structural and resting-state functional magnetic resonance imaging (fMRI) data from 10 national-level speed skaters and 14 roller skaters. Voxel-based morphometry (VBM) was used to assess whole-brain gray matter volume (GMV), and whole-brain voxel-wise resting-state analyses were conducted to calculate fractional amplitude of low-frequency fluctuations (fALFF) and degree centrality (DC).

**Results:**

Speed skaters demonstrated greater GMV in the bilateral occipital lobe, right orbitofrontal cortex, right medial temporal lobe, right insula, and cerebellum, but lower GMV in the bilateral precuneus, bilateral inferior temporal gyrus, right ventral frontal cortex, and left occipitotemporal junction compared to roller skaters (FWE corrected *p <* 0.05). Functionally, speed skaters exhibited higher DC in the left basal ganglia (caudate nucleus), while roller skaters showed higher fALFF in the right prefrontal cortex (FWE corrected *p <* 0.05).

**Conclusion:**

The pronounced structural differences may reflect distinct long-term neuroplastic characteristics related to visuomotor integration, balance 2 control, and environmental predictability inherent to each sport. The relatively limited functional differences, coupled with the common engagement of motor planning networks, may indicate a common neural feature that underlies the potential for TT. These findings provide preliminary neurobiological evidence to inform talent identification and optimize training strategies for athletes transitioning from roller skating to speed skating.

## 1 Introduction

Talent Transfer (TT) refers to the process in which athletes transition from a “donor sport” to a “transfer sport,” leveraging previously acquired skills and physical attributes to accelerate development in the new discipline (van Harten et al., 2021). This concept is of great significance in sports science, as it promotes efficient athlete development and optimal resource allocation (Rea and Lavallee, 2015). Illustrative examples include Clara Hughes, who won Olympic medals in both cycling and speed skating (Vaeyens et al., 2009; Collins and MacNamara, 2022), and Yelena Isinbaeva, who successfully transitioned from gymnastics to pole vaulting (Collins et al., 2014). The efficacy of TT is often linked to the degree of similarity between sports in terms of biomechanics, energy systems, and cognitive strategies (van Harten et al., 2021).

A quintessential example of TT is the pathway from roller skating to speed skating. Despite the different surfaces (ice vs. pavement), these sports share considerable similarities in technical movements, training methodologies, energy metabolism, and race tactics. This synergy has led to numerous international successes, such as Koen Verweij and Joey Mantia. Recognizing this potential, China formally established “dual-discipline teams” in preparation for the Beijing 2022 Winter Olympics, an initiative that culminated in a gold medal win in the men’s 500 meters event and 5th in the women’s team pursuit event.

The feasibility of TT has been discussed in relation to largely attributed to several factors, including the similarity in technical skills, training methods, energy metabolism, and competitive strategies between sports. For instance, there are significant similarities in the biomechanics and technical skills required for both sports. Both disciplines share common movements, such as gliding and turning techniques, which allows roller skaters to adapt their skills to speed skating more readily. Moreover, the training methods and energy systems employed in both sports are quite comparable. For instance, both require high levels of aerobic endurance and anaerobic power, enabling athletes to use their conditioning from roller skating to excel in speed skating. Additionally, the tactical aspects of both sports, including race strategies and pacing, are similar, providing athletes with a foundational understanding that can be applied across disciplines. This eases the transition for athletes. Despite these promising attributes, research into the neural mechanisms underpinning TT remains limited. Understanding these mechanisms could provide critical insights for enhancing talent development strategies and optimizing performance across various sports.

Researchers have employed advanced neuroimaging techniques to study the plasticity of skating athletes. For example, Wei et al. (2020) examined the relationship between volitional quality and brain morphology in elite athletes using magnetic resonance imaging (MRI) and found increased cortical thickness in the left precuneus, left inferior parietal lobe, and right superior frontal lobe, highlighting structural adaptations associated with high levels of volition (Wei et al., 2020). Zhang et al. (2021) explored neuroplasticity in elite speed skaters using resting-state functional magnetic resonance imaging (fMRI) and found increased gray matter volume (GMV) in the posterior cerebellum and frontal lobe, indicating structural adaptations from long-term training. Enhanced connectivity between the posterior cerebellum and fusiform gyrus also suggested functional changes linked to motor skills (Zhang et al., 2021). In another study, Zhang et al. (2024) examined visuospatial function in elite speed skaters. They identified heightened functional connectivity in brain regions related to attention and motor functions, indicating that extensive skating training impacts cognitive abilities (Zhang et al., 2024). These findings highlight how sustained athletic training influences both motor and cognitive processing. However, few studies have examined the brain morphological and resting-state functional characteristics of roller skaters. More studies are needed to explore these aspects and to understand the underlying neuroplasticity changes associated with these disciplines.

Given the limited research on the neural mechanisms underlying TT, this study utilized advanced fMRI to investigate brain plasticity differences between roller skating and speed skating athletes. fMRI provides high spatial resolution for precise localization of motor-related brain regions and is non-invasive, allowing safe assessment of live subjects (Moonen et al., 1990; Maguire et al., 2000). By detecting blood oxygenation level-dependent (BOLD) signals, fMRI offers insights into both structural and functional aspects of brain plasticity (Raichle et al., 2001). Recent studies indicate that long-term engagement in specific sports can lead to distinct structural and functional adaptations in the brain, particularly in regions involved in motor control, planning, and execution (Pascual-Leone et al., 2005). Roller skating and speed skating share many similarities in skating-specific movement characteristics, including gliding, turning, lower-limb coordination, and race pacing (de Boer et al., 1987). At the same time, the two sports differ in surface properties and movement constraints: roller skating relies on wheels moving over hard ground surfaces (de Boer et al., 1987), whereas speed skating requires gliding on low-friction ice. These shared and sport-specific characteristics may be reflected in both common and distinct neural features. Thus, we employed structural and resting-state functional MRI to compare brain morphology and intrinsic brain activity between national-level speed skaters and roller skaters. We hypothesized that: (1) Speed skaters would demonstrate structural adaptations in brain regions related to balance regulation and the processing of predictable visual streams (e.g., insula, cerebellum, occipital regions); (2) Roller skaters would show enhancements in areas supporting executive control and multi-sensory integration for unpredictable terrains (e.g., prefrontal cortex, precuneus); and (3) Despite these differences, shared functional networks related to core skating movements would be evident, providing preliminary neuroimaging evidence relevant to TT. Our findings aim to provide the first neurobiological insights into the “roller-to-ice” transfer, potentially informing more scientific talent identification and training frameworks.

## 2 Materials and methods

### 2.1 Participants

Ten athletes from the national speed skating team (age: 21.8 ± 2.9 years; 4 females/6 males; training experience: 10.4 ± 3.5 years) and 14 athletes from the national roller skating team (age: 17.4 ± 1.9 years; 3 females/11 males; training experience: 6.1 ± 2.9 years) participated in this study (Table 1). All participants were right-handed and had normal or corrected-to-normal vision. A screening questionnaire was administered to exclude individuals with contraindications for MRI (e.g., metal implants, history of neurological or psychiatric disorders). All participants provided written informed consent. The study was approved by the Ethics Committee of the School of Medicine at Tsinghua University (Ethics approval number: 20180016). We acknowledge that the groups were not perfectly matched in age and training experience, which is a common challenge in neuroimaging studies of elite athletes. To account for these potentials confounds, age, gender, and total years of training were included as covariates in all group-level statistical analyses.

**Table 1:** Basic demographic data of subjects.

Variable	Speed skaters (*n =* 10)	Roller skaters (*n =* 14)	Statistical test	*p*-value
Age (years)	21.80 ± 2.90	17.36 ± 1.95	t = 4.505	<0 0.001
Gender (male/female)	6/4	11/3	Fisher’s exact test	0.393
Endurance training period (year)	10.40 ± 3.50	6.14 ± 2.93	t = 3.236	0.004

Age and training years were compared using independent-samples *t*-tests. Gender distribution was compared using Fisher’s exact test.

### 2.2 Image data acquire

All MRI data were acquired using a 3.0 T Philips Achieva scanner with a 32-channel head coil. To minimize circadian effects, scans were scheduled between 9:00 and 11:00 a.m. The data collection included resting-state blood oxygen level-dependent (BOLD) imaging and T1-weighted structural imaging. To protect participants’ hearing and minimize the effects of noise on brain activity, earplugs and headphones were provided, while foam pads were used to reduce head movement within the coil. For the BOLD imaging, a single-shot echo-planar imaging sequence was applied, with a scan duration of 8 min. The parameters were as follows: TR = 2000 ms, TE = 30 ms, flip angle = 90°, 37 axial slices, slice thickness = 3 mm (with a 0.5 mm slice gap), acquisition matrix = 80 × 80, FOV = 230 × 230 mm, and voxel size = 2.87 × 2.87 × 3.50 mm^3^. During this resting-state scan, participants were instructed to stay awake, keep their eyes closed, relax, and avoid focusing on any specific thoughts. Meanwhile, for structural imaging, T1-weighted structural images were acquired with parameters set to TR = 7.5 ms, TE = 3.7 ms, flip angle = 8°, 180 slices, slice thickness = 1 mm, acquisition matrix = 232 × 230, FOV = 230 × 230 mm, and voxel size = 1 × 1 × 1 mm^3^.

### 2.3 Voxel-based morphometry analysis

The Voxel-Based Morphometry (VBM) process for analyzing the brain morphological characteristics of the two groups of athletes was conducted using the VBM8 toolbox.^1^ This method allows for an automated and unbiased assessment of brain anatomy and volume differences. The procedure involved the following rigorous steps: (1) Brain Tissue Extraction: Initial processing included isolating brain tissue from non-brain structures. This was achieved through a combination of thresholding and morphological operations to effectively remove extraneous signals, such as the skull, scalp, and other noise, ensuring that only the relevant brain tissue was retained for further analysis; (2) Normalization: The high-resolution T1-weighted images were standardized to a common spatial template using the DARTEL (Diffeomorphic Anatomical Registration Through Exponentiated Lie Algebra) method. This approach facilitates improved alignment of anatomical structures through a more accurate non-linear transformation, enhancing the robustness of spatial normalization; (3) Segmentation and Normalization to MNI Template: Following normalization, the images underwent segmentation into distinct tissue types: gray matter (GM), white matter (WM), and cerebrospinal fluid (CSF). This segmentation process is critical for isolating the gray matter, which was subsequently normalized to the MNI (Montreal Neurological Institute) template. This step ensures comparability of data across subjects and supports group-level analyses; (4) Smoothing: the modulated GM images were smoothed with an 8 mm isotropic full-width at half-maximum (FWHM) Gaussian kernel. This smoothing step was used to improve the signal-to-noise ratio, reduce residual inter-individual anatomical variability after spatial normalization, and improve the statistical properties of the data for group-level whole-brain VBM analysis. The resulting smoothed modulated GM maps were used for subsequent whole-brain gray matter volume (GMV) analyses.

### 2.4 Resting state data analysis

To investigate the brain functional plasticity of roller skaters and speed skaters, we applied resting-state data processing. The analysis of resting-state functional characteristics for the two athlete groups was conducted using the DPARSF toolbox.^2^ The procedure included preprocessing steps followed by the calculation of the fractional amplitude of low-frequency fluctuations (fALFF) and degree centrality (DC). Specifically, the steps were as follows: (1) Removal of Initial Time Points: The first five time points were removed to eliminate the effects of initial MRI signal instability; (2) Temporal correction: This step ensured synchronization of the time series data to account for any timing discrepancies during the acquisition process; (3) Spatial Correction: This involved correcting for timing differences in data acquisition within the scan and the effects of head motion. Participants exhibiting head movement exceeding 2 mm in any plane or rotation >2° in any direction were excluded from the analysis. Importantly, no subjects were eliminated during this step; (4) Segmentation and Normalization: Brain tissue was segmented into gray matter, white matter, and cerebrospinal fluid, which was then normalized to the participant’s own high-resolution T1 anatomical image. Subsequently, the data were standardized to the Montreal Neurological Institute (MNI) template and resampled to a voxel size of 3 × 3 × 3 mm^3^ to facilitate group comparisons; (5) Removal of Confounding Factors: Linear drift, global noise, and head motion parameters in 24 directions (using the Friston method) were regressed out from the time series data to enhance the accuracy of the analysis; (6) Smoothing: Finally, a Gaussian smoothing kernel with FWHM of 8 mm^3^ was applied to the images to improve the signal-to-noise ratio and prepare the data for further analysis; (7) Then, fALFF and DC were calculated. ALFF quantifies the total power of the brain’s low-frequency range, reflecting the intensity of spontaneous BOLD low-frequency amplitude. This metric, as defined by Zang et al. (2007), is widely used to detect the strength of spontaneous fluctuations in BOLD signals, with low-frequency fluctuations (0.01–0.08 Hz) containing specific physiological information (Biswal et al., 1995). In this study, we conducted a fractional amplitude of low-frequency fluctuations (fALFF) analysis, which is the ratio of low-frequency power to total frequency power (Zou et al., 2008). Furthermore, to compute the DC values, we used the pre-smoothed data to construct a binary network. DC is a common metric in resting-state brain network studies, reflecting the connectivity strength of a particular brain region with others (Bullmore and Sporns, 2009). The analysis was performed following temporal band-pass filtering (0.01 Hz < *f* < 0.08 Hz) to isolate the relevant frequency range. Each correlation was thresholded at a level of r = 0.25 (ie, sparsity set to 0.25) to generate a weighted matrix that included undirected edges. DC was then calculated based on these matrices. Fisher’s z-transformation was then applied to the DC maps to facilitate between-subject statistical comparison, and the resulting whole-brain fALFF and z-transformed DC maps were used for subsequent voxel-wise group analyses.

### 2.5 Statistical analysis

Second-level analyses of the MRI data were performed using SPM12^3^ within the general linear model (GLM) framework. For whole-brain GMV, fALFF, and DC maps, separate two-sample t-tests were conducted to assess between-group differences between speed skaters and roller skaters. Age, gender, and training years were entered as covariates in the design matrix of all second-level GLMs to reduce the potential influence of demographic and training-related differences. Gender was coded as a binary variable. The significance threshold for all between-group voxel-wise comparisons was set at *p <* 0.05, family-wise error (FWE) corrected, following established neuroimaging statistical recommendations for controlling false-positive findings in massive voxel-wise testing (Nichols and Hayasaka, 2003).

In addition to the primary FWE-corrected analyses, supplementary false discovery rate (FDR)-corrected analyses were conducted as robustness checks and are reported in the Supplementary material (Poldrack et al., 2017). Given the relatively small sample size, a post-hoc sensitivity estimate was conducted using G-Power to characterize the detectable effect size of the current two-group design, and the results are reported in the Supplementary material.

**Figure 1:**
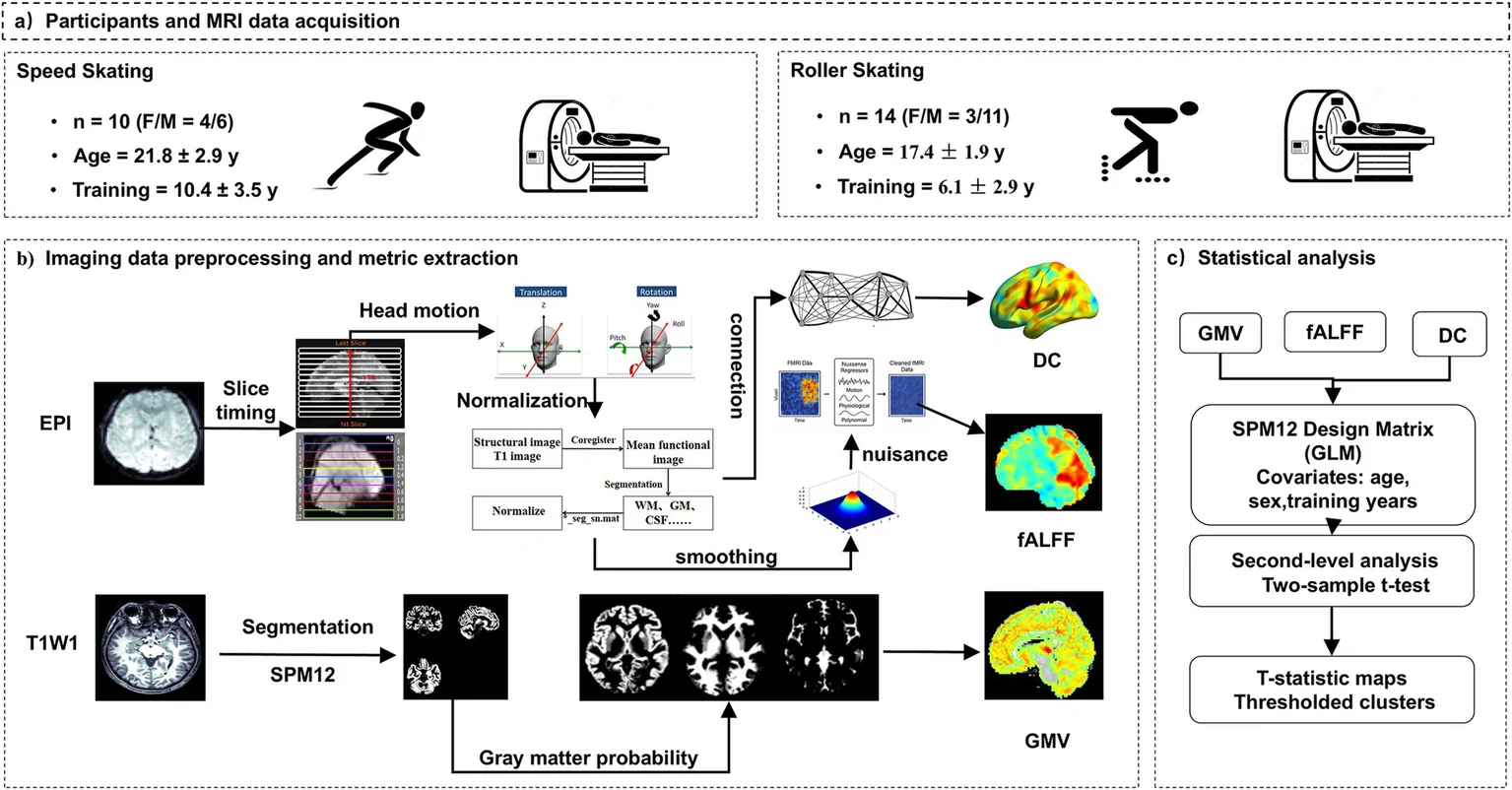
Experimental workflow and data-processing pipeline. **(a)** Participant grouping and MRI data acquisition in speed skaters and roller skaters. **(b)** Preprocessing of resting-state fMRI and T1-weighted images and computation of gray matter volume (GMV), fractional amplitude of low-frequency fluctuations (fALFF), and degree centrality (DC). **(c)** Statistical analysis using SPM12 with a general linear model including age, sex, and training years as covariates, followed by second-level two-sample *t*-tests.

The statistical analysis results were visualized using the BrainNet Viewer toolbox.^4^ This approach ensured a comprehensive evaluation of the effects of training and performance on brain structure and function across the two groups. The overall experimental workflow and data processing pipeline are summarized in Figure 1.

## 3 Results

### 3.1 Brain morphological plasticity

**Figure 2:**
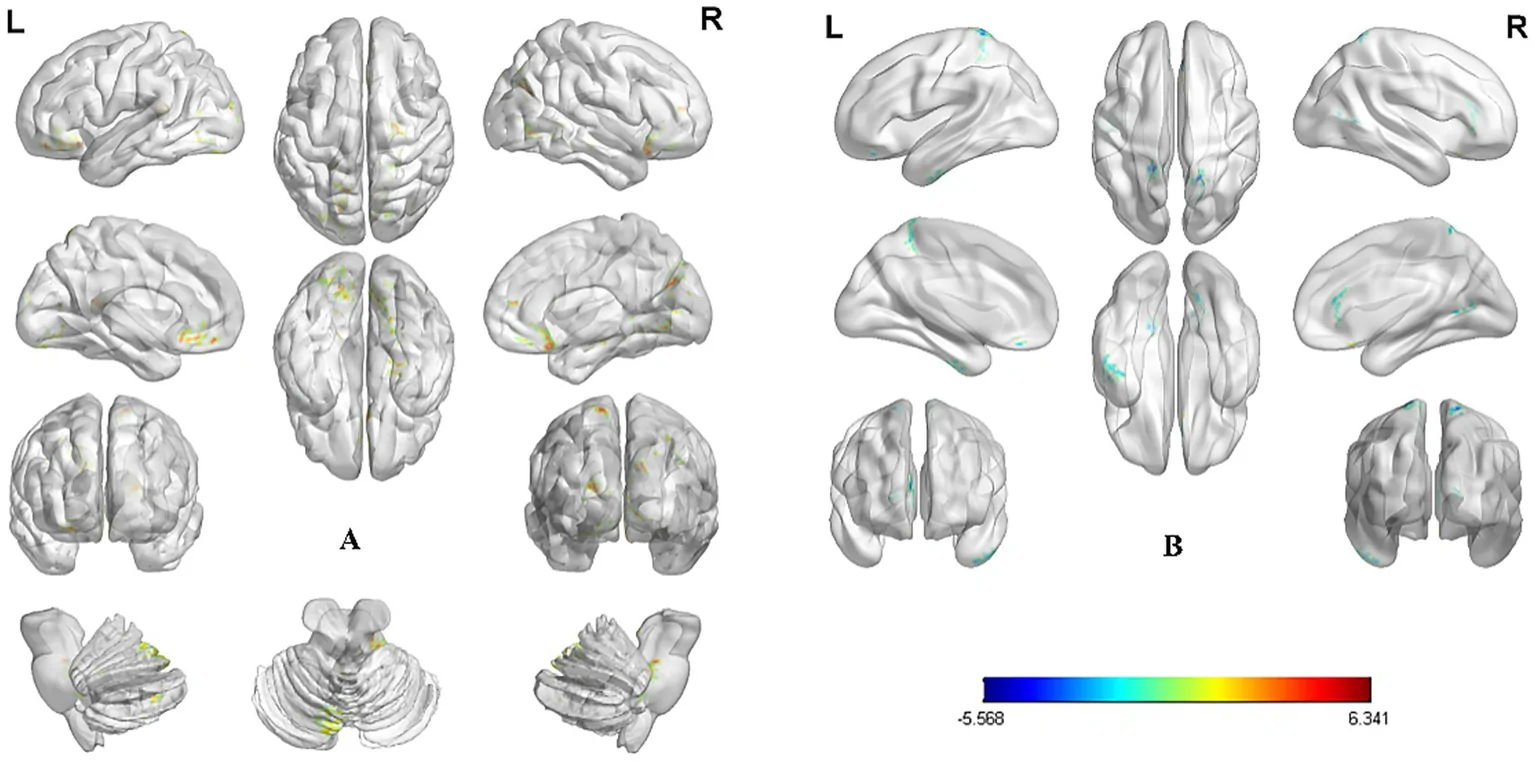
Group differences in GMV between speed skaters and roller skaters. **(A)** Regions where speed skaters showed significantly greater GMV compared with roller skaters. **(B)** Regions where roller skaters showed significantly greater GMV compared with speed skaters. Results are displayed on the standard brain surface template in multiple views. Warm colors represent higher GMV in speed skaters, while cool colors represent higher GMV in roller skaters. The color bar indicates corresponding *t*-values. Threshold: *p <* 0.05, FWE corrected.

Group comparisons revealed distinct patterns of gray matter morphology between speed skaters and roller skaters, with multiple clusters surviving FWE correction (*p <* 0.05). As shown in Figure 2 and detailed in Table 2, speed skaters exhibited significantly greater GMV in several visuomotor- and sensorimotor-related regions. These included two clusters in the left occipital lobe (cluster sizes = 374 and 729 voxels; peak T = 3.52 and 4.296; MNI = −22.5/21/−25.5 and −57/−25.5/7.5), a large cluster in the right occipital visual cortex (1760 voxels; peak T = 4.337; MNI = 54/−42/−14), and a cluster extending into the right occipitotemporal junction (peak T = 6.034; MNI = 63/−31.5/−15). Additional increases were observed in the right orbitofrontal cortex (411 voxels; peak T = 4.963; MNI = 1.5/40.5/−15), right medial temporal lobe (802 voxels; peak T = 5.197; MNI = 10.5/−66/−6), right insula (373 voxels, peak T = 4.662; MNI = 37.5/−13.5/10.5), and cerebellar regions (939 voxels, peak T = 4.658; MNI = −6/−67.5/−16.5).

**Table 2:** Brain regions with different GMV between speed skating and roller skating athletes.

Brain region	Hemisphere	Cluster size	MNI coordinate			Peak T
			x	y	z	
Occipital lobe	L	374	−22.5	21	−25.5	3.52
Occipital lobe	L	729	−57	−25.5	7.5	4.296
Occipital visual cortex	R	1760	54	−42	−14	4.337
Occipitotemporal junction	R	1760	63	−31.5	−15	6.034
Orbitofrontal cortex	R	411	1.5	40.5	−15	4.963
Medial temporal lobe	R	802	10.5	−66	−6	5.197
Insula	R	373	37.5	−13.5	10.5	4.662
Cerebellum		939	−6	−67.5	−16.5	4.658
Precuneus	L	346	−12	−54	72	−5.054
Precuneus	R	326	16.5	−52.5	69	−4.749
Inferior temporal gyrus	L	614	−49.5	−15	−37.5	−4.354
Inferior temporal gyrus	R	1779	18	−40.5	−4.5	−5.497
Ventral frontal cortex	R	335	16.5	40.5	9	−4.576
Occipitotemporal junction	L	1779	−16.5	−102	−15	−5.353

*p <* 0.05, FWE correction. R = right, L = left. A positive value of the peak point T indicates that the GMV of the speed skating group is higher than that of the roller skating group, and vice versa, it indicates that the GMV of the skating group is lower than that of the roller skating group. The coordinates of the activation point are shown in MNI coordinates.

In contrast, roller skaters showed greater GMV than speed skaters in several regions associated with visuospatial integration and higher-order perceptual processing. These included bilateral precuneus clusters (346 and 326 voxels; peak T = −5.054 and −4.749; MNI = −12/−54/72 and 16.5/−52.5/69) and bilateral inferior temporal gyrus (614 and 1779 voxels; peak T = −4.354 and −5.497; MNI = −49.5/−15/−37.5 and 18/−40.5/−4.5). Additional reductions in speed skaters were observed in the right ventral frontal cortex (335 voxels; peak T = −4.576; MNI = 16.5/40.5/9) and left occipitotemporal junction (peak T = −5.353; MNI = −16.5/−102/−15).

Together, these spatially distributed clusters illustrate a robust pattern of sport-specific structural plasticity, consistent with the regional differences shown in Figure 2. Supplementary FDR-corrected analyses of GMV showed a spatial pattern consistent with the primary FWE-corrected findings and are reported in Supplementary Table S2.

### 3.2 Resting-state functional plasticity

**Figure 3:**
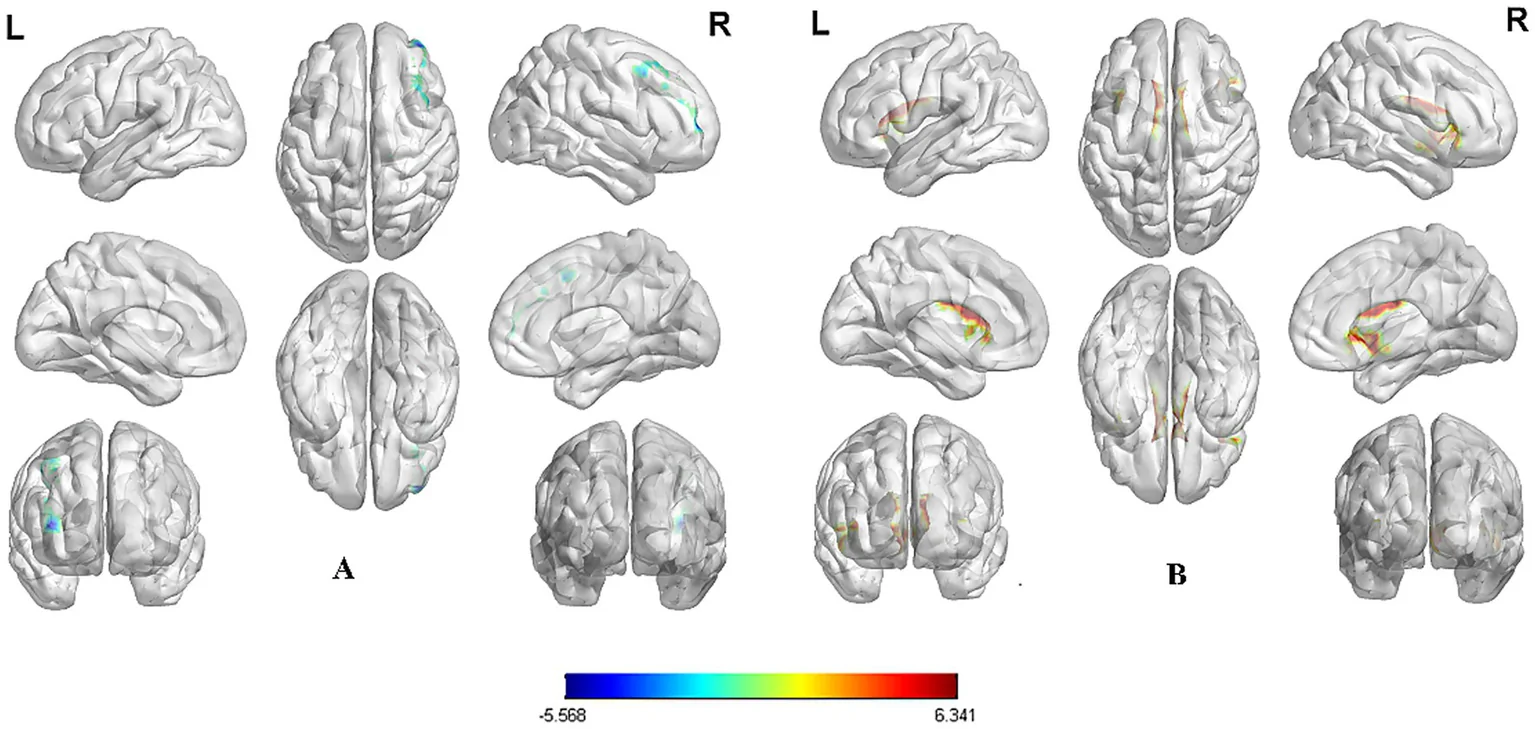
Resting-state functional differences between speed skaters and roller skaters. 1 **(A)** Regions where speed skaters showed significantly lower fALFF compared with roller skaters. **(B)** Regions where speed skaters showed significantly higher DC compared with roller skaters. Results are displayed on the standard brain surface template. Warm colors indicate greater values in roller skaters, while cool colors indicate greater values in speed skaters. The color bar represents corresponding *t*-values. Threshold: *p <* 0.05, FWE corrected.

Compared with the widespread structural differences, resting-state functional metrics revealed a more restricted set of group differences (FWE corrected, *p <* 0.05). As shown in Figure 3 and detailed in Table 3, speed skaters demonstrated significantly higher degree centrality (DC) in the left basal ganglia, primarily encompassing the caudate nucleus (cluster size = 679 voxels; peak T = 6.341; MNI = −15/24/12). This cluster reflects enhanced functional connectedness within a key node of the motor control and action-selection network. Conversely, roller skaters exhibited significantly greater fractional amplitude of low-frequency fluctuations (fALFF) in the right prefrontal cortex (cluster size = 944 voxels; peak T = −5.568; MNI = 15/36/42). The cluster extended across regions corresponding to the premotor and dorsolateral prefrontal cortices, indicating stronger spontaneous low-frequency activity in these areas relative to speed skaters. Because both fALFF and DC were examined using whole-brain voxel-wise group comparisons, under the same FWE-corrected threshold, no suprathreshold fALFF cluster was observed in the caudate nucleus, and no suprathreshold DC cluster was observed in the prefrontal cortex.

**Table 3:** Brain regions with different DC and fALFF between speed skating and roller skating athletes.

Brain region	Hemisphere	Cluster size	MNI coordinate			Peak T
			x	y	z	
DC						
Basal ganglia (caudate nucleus)	L	679	−15	24	12	6.341
fALFF						
Prefrontal cortex	R	944	15	36	42	−5.568

*p <* 0.05, FWE correction. R = right, L = left. A positive value of the peak point T indicates that the DC of the speed skating group is higher than that of the roller skating group, and vice versa, it indicates that the fALFF of the skating group is lower than that of the roller skating group. The coordinates of the activation point are shown in MNI coordinates.

No additional clusters survived FWE correction for either functional metric. Together, these results suggest that functional differences between the two groups are more focal, involving selective alterations in motor-planning and executive-control regions, as visualized in Figure 3. Supplementary FDR-corrected analyses of functional metric showed a pattern consistent with the primary FWE-corrected findings and are reported in the Supplementary Table S3.

## 4 Discussion

This study represents the first attempt to compare structural and resting-state functional cerebral characteristics between speed skaters and roller skaters, aiming to provide preliminary neuroimaging evidence relevant to “roller-to-ice” TT from the perspective of central nervous system plasticity. The findings revealed relatively limited differences in resting-state functional patterns but more pronounced structural differences, suggesting that possible partial functional overlap may coexist with sport-associated structural characteristics. These results provide two important implications. First, for talent identification, they highlight potential candidate neural features that could inform the selection and training of speed skating athletes from the roller skating population. Second, for training optimization, they suggest that targeted, sport-specific interventions are necessary to improve the success rate of cross-discipline transitions. Taken together, the present findings contribute to advancing “roller-to-ice” training systems from empirical practices toward more scientific and evidence-based strategies.

### 4.1 Functional similarity as a foundation for transfer

Although roller skating and speed skating differ in many different aspects, this study observed only minor differences in resting-state brain functional plasticity between the two sports. Differences were observed only in limited brain regions. Specifically, speed skaters showed greater DC in the left basal ganglia (caudate nucleus), a region critically involved in motor planning, sequential coordination, and automatization of movement patterns (Parsons et al., 2005; Jankowski et al., 2009). This enhancement may reflect the greater demand for anticipatory timing and rhythmic weight-shifting required on ice. By contrast, roller skaters exhibited higher fALFF in the right prefrontal cortex, including premotor and dorsolateral prefrontal regions, which are closely linked to executive control, motor cognition, and adaptive regulation of effort (Miller and Cohen, 2001; Thompson-Schill et al., 2005). This may reflect the cognitive and attentional resources needed to adapt to variable ground surfaces and fluctuating environmental conditions. Previous research shows resting state functional differences between athletes from different sports under comparable or even stricter correction thresholds. For example, aerobic athletes exhibited higher fALFF in the prefrontal cortex, posterior parietal cortex, and basal ganglia (thalamus) compared with anaerobic athletes, who in turn showed greater fALFF in the posterior cerebellum (FWE *p <* 0.01) (Zhang et al., 2022). Similarly, wrestlers demonstrated enhanced functional connectivity in the left superior temporal gyrus, left parahippocampal gyrus, left orbitofrontal gyrus, and right superior–medial frontal regions compared with handball players (FWE *p <* 0.05) (Sahin Ozarslan and Duru, 2025). Moreover, our previous study revealed that elite skaters exhibited widespread alterations in resting-state networks related to visuospatial attention compared with non-athlete controls, highlighting profound neural reorganization induced by long-term specialized training (Zhang et al., 2024). The limited functional differences observed between groups may be attributable to several factors: (1) the resting-state paradigm might not fully capture sport-specific neural dynamics that are more evident during task performance; (2) both sports share fundamental sensorimotor and cognitive demands, leading to overlapping functional networks; and (3) the conservative FWE correction and limited sample size might have reduced sensitivity to detect subtle differences. Future studies employing task-based fMRI, larger samples, longitudinal designs, or more sensitive network-based statistics may help uncover finer functional distinctions. Taken together, these findings provide exploratory neurobiological evidence that speed skating and roller skating may share partially overlapping resting-state functional characteristics. This possible overlap may be relevant to “roller-to-ice” TT and provides a useful basis for future studies to examine how shared and sport-specific neural features are associated with athletes’ transition from roller skating to speed skating.

### 4.2 Structural divergence in sport-associated cerebral characteristics

In contrast to the functional findings, the study revealed pronounced structural differences, highlighting the impact of long-term, sport-specific training on brain morphology. Morphological results indicate that athletes in the skating group exhibit greater GMV in the right medial temporal lobe, bilateral occipital lobes, right occipitotemporal junction, right orbitofrontal cortex, right insula, and left cerebellum compared to those in the speed roller skating group. Conversely, the GMV in the bilateral fusiform gyrus, left temporoparietal junction, and bilateral occipital lobes is significantly lower than that of the roller skating athletes. Combining recent findings in sports psychology, it has been discovered that the morphological differences in athletes’ brains are related to various sports perception functions, which in turn influence the feedback neural mechanisms during training (Scott, 2004; Maffei et al., 2017).

The temporal lobe is associated with visual motion perception and scene analysis: research has indicated that activation of the temporal lobe reflects an increased demand for visual scene processing (Cross et al., 2006). When elite archers focus on their targets, there is significant activation in the temporal lobe regions (Kim et al., 2008). Other studies have found that the occipitotemporal cortex is involved in target representation and the planning of complex, goal-directed movements (Grill-Spector et al., 2004; Gallivan et al., 2015). In the context of speed skating, athletes must continuously process rapidly changing visual scenes, such as ice trajectories, opponent positioning, and pacing cues, and translate this information into precise motor plans. The occipital cortex is related to visual processing. Research has also shown that professional divers have a significantly thicker ventromedial prefrontal cortex compared to the general population (Wei et al., 2011). Therefore, the observed adaptations in temporal and occipitotemporal regions may be consistent with the sport-specific demand for integrating visual motion cues with anticipatory action planning, which is critical for maintaining speed and efficiency on ice.

The GMV in the right insula is associated with athletic ability and the integration of motor sensations. Research has indicated that the gray matter density of the right insula is closely related to aerobic capacity (Peters et al., 2009). It is also associated with the regulation of increased heart rate and blood pressure during the initial phase of exercise, thereby enhancing athletic performance (Wang et al., 2013). The insula is capable of extracting significant salient stimuli from multiple inputs (Menon and Uddin, 2010). It plays a crucial role in sensory-motor integration through strong coupling with the pre-motor area, sensory-motor area, supplementary motor area, and the cingulate gyrus (Wang et al., 2013). Additionally, research has found that the insula is crucial for gymnasts, primarily through its role in integrating the athlete’s bodily sensations with their environment and coordinating the various parts of the body (Kim et al., 2008). Simmons et al. (2012) suggest that the athlete’s insula allows for highly accurate predictions of bodily sensations in the next moment. In speed skating, where balance regulation, interoceptive awareness, and continuous postural adjustments are essential on low-friction ice, these insular differences may be associated with the sport-specific requirements to integrate internal bodily cues with external environmental demands, ultimately supporting stability and performance efficiency.

By contrast, roller skaters demonstrated greater GMV in the bilateral precuneus, inferior temporal gyrus, right ventral frontal cortex, and left occipitotemporal junction. These regions are strongly associated with visuospatial processing, proprioceptive integration, and executive control. For example, the precuneus, located in the parietal lobe, plays a central role in integrating somatosensory (proprioceptive) and visual information to guide posture and adjust movement in dynamic environments (Cavanna and Trimble, 2006; Kim et al., 2008). The inferior temporal and occipitotemporal cortices support high-level visual recognition and help process complex visual information, which may support athletes in adapting to variable terrains and rapidly changing surroundings (Gu et al., 2021). In addition, the ventral frontal cortex contributes to executive functions such as action monitoring and flexible decision-making, which are crucial in situations that require rapid tactical adjustments (Acuña et al., 2010). Taken together, these structural adaptations may reflect the sport-specific perceptual and cognitive demands of roller skating, in which athletes must constantly adjust posture, integrate external visuospatial information, and exercise strategic control to cope with the variability of land-based training and competition environments.

From the perspective of “roller-to-ice” TT, these structural findings may help clarify how previously developed neural characteristics in roller skaters could be used and further refined during the transition to speed skating. Previous talent-transfer studies have emphasized that successful transition is not determined solely by similarity between the donor and target sports, but also by how athletes adapt previously acquired capacities to the demands of the new sport context (MacNamara and Collins, 2015; van Harten et al., 2021; Green et al., 2024). The greater GMV observed in roller skaters in the bilateral precuneus, inferior temporal gyrus, right ventral frontal cortex, and left occipitotemporal junction may reflect neural characteristics supporting visuospatial monitoring, environmental updating, high-level visual processing, and flexible action control in land-based skating environments. These characteristics may provide useful pre-existing resources during the early stage of adaptation to ice skating, because athletes can draw on perceptual and motor-control capacities developed through roller skating. At the same time, the structural divergence between roller skaters and speed skaters indicates that these capacities may need to be further refined and integrated to meet the rhythm-dependent, low-friction, and technically constrained demands of skating on ice. Thus, the structural findings may not only reflect sport-specific neural characteristics but also help identify functional domains that require targeted refinement during roller-to-ice transition training.

### 4.3 Practical implications for talent identification and training

This study’s findings are important for TT from roller skating to speed skating. From athlete selection perspective, structural changes in brain regions are promising. These areas include the cerebellum, insula, and occipitotemporal cortex. They support balance, body awareness, and vision-movement coordination. These neural features can serve as new potential reference markers. When combined with traditional measures, they may help identify skaters with high potential for switching to ice. This method makes selection more evidence-based and less intuitive. It improves both efficiency and fairness in choosing athletes across sports.

From training perspective, shared brain networks and sport-specific changes require gradual training. Early transition should focus on land-based balance practice. Examples include single-leg stands and simulated ice kicks. These activate cerebellum and insula circuits. They help athletes adapt to ice instability. Later, rhythmic on-ice drills like timed weight-shifting can be used. These use basal ganglia circuits to build movement automaticity. Differences in occipitotemporal areas also show need for vision-action retraining. Examples are trajectory tracking or visual feedback drills. These improve sensorimotor integration in complex environments.

Overall, these results support a new training framework for roller-to-ice transition. This framework is based on neuroplasticity. It first builds neural adaptations off ice. Then, it moves skills to on-ice settings. This approach improves training efficiency. It also shows how neuroscience can modernize cross-sport training. Combining selection and training with neural principles can increase success in TT. It helps develop more elite speed skaters sustainably.

### 4.4 Limitations and future directions

This study has several limitations that should be acknowledged. First, the sample size was relatively small (10 speed skaters and 14 roller skaters), which may have reduced the statistical power of the whole-brain VBM and resting-state functional analyses, limited the stability of the findings, and increased the risk of both false-positive and false-negative results. This was primarily due to the challenge of recruiting elite national-level athletes. Although the primary findings survived FWE correction and the supplementary FDR-corrected analyses showed a consistent spatial pattern, the post-hoc sensitivity estimate indicated that the present design was mainly sensitive to large between-group effects. Therefore, small-to-moderate neural differences may have remained undetected. Second, the two groups were not fully matched in age and training experience. Although age, gender, and training years were included as covariates in the second-level GLM, covariate adjustment cannot fully compensate for incomplete group matching in a small and unbalanced sample. In addition, the cross-sectional design prevents causal inference, and the lack of behavioral or performance measures limits direct interpretation of how the observed neural differences relate to actual skating performance or transition success. Future studies should include larger, better-matched samples and incorporate longitudinal, behavioral, physiological, and performance assessments. Consequently, we cannot establish a direct link between the observed neural differences and actual athletic performance or skill transfer efficacy. Third, the resting-state functional findings should be interpreted cautiously. Although both fALFF and DC were examined using whole-brain voxel-wise analyses, the significant effects were observed in different metrics and regions, and a formal group × region × metric interaction was not tested. Moreover, the relatively limited functional differences may reflect partial functional overlap, but may also be influenced by limited statistical power or the sensitivity limits of resting-state metrics. Finally, sleep duration, caffeine intake, and recent physical activity before scanning were not systematically recorded. Future studies should better standardize these factors and test whether targeted interventions can support roller-to-ice transition outcomes.

In conclusion, this study provides preliminary neuroimaging evidence that speed skaters and roller skaters show both partially overlapping resting-state functional characteristics and distinct structural brain adaptations associated with their specific sport demands. This neural profile offers a valuable perspective for understanding “roller-to-ice” talent transfer. The relatively limited functional differences may reflect shared sensorimotor and cognitive features between the two skating disciplines, while the structural differences highlight sport-associated neural characteristics that may both support early adaptation to ice skating and indicate functional domains requiring further refinement during transition training. By integrating neuroimaging findings with traditional coaching assessments, future research may help develop a more scientific and evidence-informed framework for identifying and supporting athletes during the transition from roller skating to speed skating.

## Data Availability

The original contributions presented in the study are included in the article/Supplementary material, further inquiries can be directed to the corresponding author.

## Footnotes

1
http://dbm.neuro.uni-jena.de/vbm

2
http://www.restfmri.net

3
https://www.fil.ion.ucl.ac.uk/spm/software/download/

4
https://www.nitrc.org/frs/?group_id=504
