# Correction: Cerebral differences between roller and speed skaters: preliminary evidence for roller-to-ice talent transfer

**DOI:** 10.3389/fpsyg.2026.2029951

**Published:** 2026-10-27

**Authors:** Qing Yan, Keying Zhang, Ling Jiang, Yuyan Wang, Yihua Cao, Chunmei Cao, Dong Zhang

**Affiliations:** 1 Institute of Artificial Intelligence in Sports, Capital University of Physical Education and Sports, Beijing, China; 2 Department of Physical Education, Southeast University, Nanjing, China; 3 Division of Sports Science and Physical Education, Tsinghua University, Beijing, China

**Keywords:** brain plasticity, magnetic resonance imaging, neural mechanism, skating, talent transfer

The following statement should be added to the **Acknowledgments** section: “The authors would like to acknowledge the support of the Beijing Key Laboratory of Interdisciplinary Intelligent Technologies in Sports Medicine and Engineering.”

The original version of this article has been updated.

