# Supplementary material for: Cerebral differences between roller and speed skaters: preliminary evidence for roller-to-ice talent transfer
Source: Front Psychol. 2026 Jul 17;17:1778839. doi: 10.3389/fpsyg.2026.1778839 (PMC13424456; doi:10.3389/fpsyg.2026.1778839)
Supplement: Supplementary file 1 [file Supplementary_file_1.DOCX]

**Supplementary Material**

Cerebral Differences Between Roller and Speed Skaters: Preliminary Evidence for Roller-to-Ice Talent Transfer

**Table S1.** Approximate peak-level effect-size estimates for significant clusters..................2

**Table S2**.FDR-corrected results for GMV group comparisons.............................................4

**Table S3.**FDR-corrected results for DC and fALFF group comparisons..............................5

**Supplementary Table S1**. Approximate peak-level effect-size estimates for significant clusters

| **Metric** | **Brain region** | **Hemisphere** | **Cluster size** | **Peak T** | **Cohen’s d** |
| --- | --- | --- | --- | --- | --- |
| GMV | Occipital lobe | L | 374 | 3.520 | 1.46 |
| GMV | Occipital lobe | L | 729 | 4.296 | 1.78 |
| GMV | Occipital visual cortex | R | 1760 | 4.337 | 1.80 |
| GMV | Occipitotemporal junction | R | 1760 | 6.034 | 2.50 |
| GMV | Orbitofrontal cortex | R | 411 | 4.963 | 2.05 |
| GMV | Medial temporal lobe | R | 802 | 5.197 | 2.15 |
| GMV | Insula | R | 373 | 4.662 | 1.93 |
| GMV | Cerebellum | — | 939 | 4.658 | 1.93 |
| GMV | Precuneus | L | 346 | -5.054 | 2.09 |
| GMV | Precuneus | R | 326 | -4.749 | 1.97 |
| GMV | Inferior temporal gyrus | L | 614 | -4.354 | 1.80 |
| GMV | Inferior temporal gyrus | R | 1779 | -5.497 | 2.28 |
| GMV | Ventral frontal cortex | R | 335 | -4.576 | 1.89 |
| GMV | Occipitotemporal junction | L | 1779 | -5.353 | 2.22 |
| DC | Basal ganglia / caudate nucleus | L | 679 | 6.341 | 2.63 |
| fALFF | Prefrontal cortex | R | 944 | -5.568 | 2.31 |

| **Brain region** | **Hemisphere** | **Cluster size** | **MNI coordinate** | | | **Peak T** | **Cluster p(FDR-corr)** |
| --- | --- | --- | --- | --- | --- | --- | --- |
|  |  |  | **x** | **y** | **z** |  |  |
| Occipital lobe | L | 374 | -22.5 | 21 | -25.5 | 3.52 | 0.002 |
| Occipital lobe | L | 729 | -57 | -25.5 | 7.5 | 4.296 | <0.001 |
| Occipital visual cortex | R | 1760 | 54 | -42 | -14 | 4.337 | <0.001 |
| Occipitotemporal junction | R | 1760 | 63 | -31.5 | -15 | 6.034 | <0.001 |
| Orbitofrontal cortex | R | 411 | 1.5 | 40.5 | -15 | 4.963 | 0.002 |
| Medial temporal lobe | R | 802 | 10.5 | -66 | -6 | 5.197 | <0.001 |
| Insula | R | 373 | 37.5 | -13.5 | 10.5 | 4.662 | 0.002 |
| Cerebellum |  | 939 | -6 | -67.5 | -16.5 | 4.658 | <0.001 |
| Precuneus | L | 346 | -12 | -54 | 72 | -5.054 | 0.011 |
| Precuneus | R | 326 | 16.5 | -52.5 | 69 | -4.749 | 0.011 |
| Inferior temporal gyrus | L | 614 | -49.5 | -15 | -37.5 | -4.354 | <0.001 |
| Inferior temporal gyrus | R | 1779 | 18 | -40.5 | -4.5 | -5.497 | <0.001 |
| Ventral frontal cortex | R | 335 | 16.5 | 40.5 | 9 | -4.576 | 0.011 |
| Occipitotemporal junction | L | 1779 | -16.5 | -102 | -15 | -5.353 | <0.001 |

**Table S2.** FDR-corrected results for GMV group comparisons

**Table S3.FDR-corrected results for DC and fALFF group comparisons**

| **Brain region** | **Hemisphere** | **Cluster size** | **MNI coordinate** | | | **Peak** | **Cluster p(FDR-corr)** |
| --- | --- | --- | --- | --- | --- | --- | --- |
|  | | | **x** | **y** | **z** |  | |
| **DC** | | | | | | | |
| Basal ganglia (caudate nucleus) | L | 679 | -15 | 24 | 12 | 6.341 | 0.002 |
| **fALFF** | | | | | | | |
| Prefrontal cortex | R | 944 | 15 | 36 | 42 | -5.568 | <0.001 |
